# A structural study of PrCrO_3_ under extreme conditions: a comparison with the effects of doping

**DOI:** 10.1098/rsta.2022.0332

**Published:** 2023-10-16

**Authors:** C. L. Bull, N. P. Funnell, C. J. Ridley

**Affiliations:** ^1^ ISIS Neutron and Muon Facility, STFC, Rutherford Appleton Laboratory, Chilton, Didcot OX11 0QX, UK; ^2^ School of Chemistry, University of Edinburgh, David Brewster Road, Edinburgh EH9 3FJ, UK

**Keywords:** structure, distortion, polyhedral-tilting, orthochromites, lanthanide-perovskites

## Abstract

The nuclear and magnetic structure of ${\text{PrCrO}}_{3}$ has been investigated using neutron and X-ray powder diffraction as a function of pressure and temperature. The orthorhombic symmetry (space group Pbnm) remains stable up to the highest temperature (1500 K) and pressure (approx. 6 GPa) considered. There is a crossover in the magnitude of the *a-* and *b*-lattice parameters at approximately 1135 K, caused by competing effects of octahedral tilting and distortion. The material is antiferromagnetic ($T_{\text{N}}\approx 240$ K) with $Pb^{\prime }n^{\prime }m$ symmetry, with a maximum moment of $2.34(2)\mu_{b}$ on the ${\mathrm{Cr}}^{3+}$ sites aligned along the direction of the a-axis. The application of pressure shows an abnormal softening in the unit-cell volume, which is suggestive of a continuous approach to a second-order phase transition. Raman spectroscopy measurements at ambient temperature were collected as a function of pressure up to approximately 12 GPa, with discontinuous mode behaviour further suggesting the existence of a transition above 7 GPa. The measured structural changes in ${\text{PrCrO}}_{3}$ are compared extensively in the wider context of other lanthanide orthochromites, and the comparative effects of *A*- and *B*-site substitution on the polyhedral tilts and distortion are discussed.

This article is part of the theme issue ‘Exploring the length scales, timescales and chemistry of challenging materials (Part 1)’.

## 1. Introduction

As a result of chemical doping, or the variation of temperature or pressure, perovskite-like materials (with general chemical formula ${\text{ABX}}_{3}$) can adopt a wide range of structural distortions away from the aristotype cubic structure (parent cell). The structure may be altered via three routes: (a) distortion of the ${\text{BO}}_{6}$ octahedra (for example, a Jahn–Teller distortion), (b) a tilt (or rotation) of the rigid ${\text{BX}}_{6}$ octahedral units around the principal axes of the parent cell [1] and (c) *A*-site cation displacements [2].

These distortion mechanisms drive many of the resultant electronic, magnetic, and other physical properties of these materials, and have been studied extensively in the literature; a full review of which is beyond the scope of this study. Here, we present a structural study of ${\text{PrCrO}}_{3}$, one of a series of orthorhombic perovskites (with the general formula ${\text{RCrO}}_{3}$ where R=rare earth) which show complex magnetic behaviour at low temperature and interesting magnetoelectric and multiferroic properties [3]. The structural distortions of these materials can be driven further towards higher/lower symmetry structures using extreme conditions, such as high pressures and temperatures. This can be understood using Landau theory, while the relative raising or lowering of symmetry can be predicted using qualitative methods [4].

The semiconducting orthorhombic ${\text{PrCrO}}_{3}$ (with Cr in the +3 oxidation state) has been shown by optical methods to have an estimated band gap in the range of 3.20–3.26 eV and as a result is a good candidate for opto-electronic devices [5]. Magnetically, the material is antiferomagnetic with a Néel temperature of 237 K. At 4.2 K, the ${\mathrm{Cr}}^{3+}$ spin structure is G-type with an additional weak ferromagnetic component laying along the c-axis [6]. Permittivity and impedance spectroscopic measurements suggest that the material may be relaxor ferroelectric-like in nature [7]. Very few structural studies have been performed on the material to date [3,8]. We have studied the structural behaviour of ${\text{PrCrO}}_{3}$ using a combination of neutron and X-ray diffraction, both as a function of pressure and temperature. We have also investigated the behaviour of the Raman active modes where for some vibrational modes softening is found to occur across the pressure range studied.

## 2. Experimental

### (i) Sample synthesis

${\text{PrCrO}}_{3}$ was prepared by mixing stoichiometric quantities of dried ${\text{Pr}}_{6}{\text{O}}_{11}$ and ${\text{Cr}}_{2}{\text{O}}_{3}$ (Sigma–Aldrich >99%) [3]. The ground powder was pelletized prior to annealing at 923 K for 12 h in a Pt crucible. The annealed pellet was reground and re-pelletized and annealed for a further 24 h at 1223 K and this process repeated at 1623 K [3]. The sample was then characterized by X-ray diffraction using a Bruker D2 diffractometer to confirm phase purity.

### (ii) Ambient-pressure diffraction

The ambient pressure, ambient temperature structure was confirmed using the Polaris instrument at the ISIS Neutron and Muon Source, UK [9]. Low-temperature neutron powder diffraction data were collected using the GEM instrument at the ISIS Neutron and Muon Source [10]. Approximately 2 g of ${\text{PrCrO}}_{3}$ was dried under vacuum at 390 K before being loaded into a 6 mm vanadium canister in an inert atmosphere, sealed using indium. The sample was cooled to 10 K, allowed to reach equilibrium for 15 min, and data were collected on warming the sample in 10 K steps up to 100 K, and 20 K steps from 100 to 280 K. The sample was allowed 5 min to reach equilibrium at each temperature, before starting the measurement. Each temperature point was measured for approximately 1 h. High-temperature powder X-ray diffraction (PXRD) measurements were collected using a Rigaku Smartlab with a Cu Kα monochromator. The sample was loaded into a 0.8 mm deep alumina sample holder, and mounted onto the high-temperature stage. Data were collected over the range $20^{\circ }<2\theta <65^{\circ }$ for approximately 1 h per measurement. The sample was heated in 100 K steps, with data collected on heating, allowing for 15 min to reach equilibrium at each point. Symmetry-adapted basis vector decomposition was performed using ISODISTORT [11], the resultant modes can be visualized using the associated ISOVIZ program. Representative parent and distorted cif structure files, and the resultant ISOVIZ visualization file, are included as part of the electronic supplementary material.

### (iii) High-pressure neutron powder diffraction

High-pressure neutron powder diffraction measurements were performed on the PEARL instrument at the ISIS Neutron and Muon Source, UK [12]. Powdered ${\text{PrCrO}}_{3}$ was loaded into a null-scattering TiZr gasket. Pb was included as a pressure-marker, and perdeuterated methanol : ethanol (4 : 1 by volume) was used as a pressure medium. The gasket was compressed using single-toroid ${\text{ZrO}}_{2}/{\text{Al}}_{2}{\text{O}}_{3}$ anvils within a V3 Paris–Edinburgh press [13]. Diffraction patterns were measured in the fixed $2\theta =90^{\circ }$ scattering geometry, for approximately 2 h per pressure point. The raw data were corrected for detector efficiency and anvil attenuation using the Mantid software [14]. Rietveld refinement was performed using the GSAS suite of programs [15]. The bulk modulus and axial compressibilties were determined using the *PASCal* program [16].

### (iv) High-pressure Raman spectroscopy

High-pressure Raman measurements were performed using a Merrill–Basset diamond anvil cell [17]. A stainless steel gasket was preindented from an initial thickness of 200 μm to 75 μm, and drilled with a sample hole of 250 μm diameter. A small ruby sphere was used as pressure marker, determined by ruby fluorescence measurements [18]. Methanol : ethanol (4 : 1 by volume) was used as a pressure transmitting medium. Raman spectra were collected using an in-house Raman system equipped with a Princeton Instruments SP2500i spectrometer using a 1800 g holographic blaze grating. A diode laser (λ=532.23 nm) was focused using a 20× Mitutoyu objective lens.

## 3. Results and discussion

### (a) Ambient-pressure crystallographic structure

**Figure 1.**
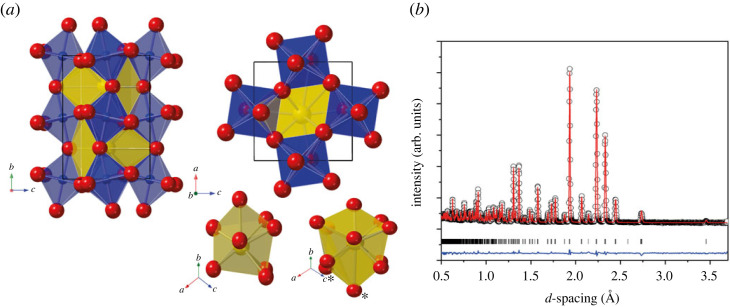
(*a*) Structure of ${\mathrm{PrCrO}}_{3}$ under ambient conditions. The ${\mathrm{CrO}}_{6}$ octahedral units are shown in blue with oxygen atoms (red) at the shared corners. The ${\mathrm{PrO}}_{n}$ polyhedral units are shown in yellow. The isolated ${\mathrm{PrO}}_{n}$ units are shown below the main structure, for n=8 (lower-left) and n=12 (lower-right). The more obvious additional oxygen atoms for n=12 are indicated by an asterisk and the remaining additional oxygen atoms are behind other more visible oxygen atoms. (*b*) Ambient pressure/temperature neutron diffraction pattern and Rietveld refinement of *Pbnm* structure of ${\mathrm{PrCrO}}_{3}$. Data collected from Polaris $2\theta =90^{\circ }$ detector banks. The collected data are shown as open black circles, the Rietveld fit as the red trace and the residual of the fit to the data as the blue trace. The vertical tick marks show the expected reflection positions for the orthorhombic unit cell described in the main text. (Online version in colour.)

Figure 1 shows the neutron diffraction pattern (and Rietveld refinement) for powdered ${\text{PrCrO}}_{3}$ collected under ambient conditions using the Polaris instrument. The derived structural parameters from the Rietveld fit are given in table 1. The orthorhombic (Pbnm) structure is that of a distorted perovskite, Glazer tilt $a^{-}a^{-}c^{+}$, where the ${\text{CrO}}_{6}$ octahedra are tilted away from the ideal cubic structure via the R4+ and M3+ primary distortion modes ($Pm\bar{3}m$ parent with *B*-site at the origin, [a+b,b−a,2c]; ($\frac{1}{2},\frac{1}{2},0$)) [1,19]. A schematic of these distortion modes is available in the electronic supplementary material. Although tilted, the ${\text{CrO}}_{6}$ octahedra are regular with the Cr–O distances ranging from 1.972 to 1.982 Å with a weighted average value of 1.976 Å, see figure 1. The Pr–O–Pr bond angles along the chains of the ${\text{CrO}}_{6}$ octahedra are $155^{\circ }$, and the resultant tilts are calculated to be $8.82^{\circ }$ for the anti-phase $a^{-}$ (R4+ mode), and $8.73^{\circ }$ for the in-phase $c^{+}$ (M3+ mode) at ambient conditions.

**Table 1.** Refined structural parameters, and resulting bond lengths, for ${\mathrm{PrCrO}}_{3}$ from neutron powder diffraction at 10 K (GEM), and 300 K (Polaris). The symmetry is Pbnm with Pr and O1 sitting on the *4c* ($x,y,\frac{1}{4}$), Cr sitting on the *4b* ($\frac{1}{2},0,0$), and O2 on the *8d* (x,y,z) Wyckoff sites, respectively. The bond lengths for Pr are shown assuming eightfold coordination to O, with 2× distinct Pr-O1 bonds and 3× distinct Pr-O2 bonds. The additional bond lengths assuming 12-fold coordination to O are shown below in square brackets.

temperature (K)	10	300
a-axis length (Å)	5.4435(3)	5.45346(6)
b-axis length (Å)	5.4759(4)	5.47864(7)
c-axis length (Å)	7.7003(4)	7.71796(8)
unit-cell volume (${\text{Å}}^{3}$)	229.53(2)	230.593(5)
Pr x,y	−0.0093(8),0.0369(4)	−0.0065(3),0.03591(13)
O(1) x,y	0.0772(5), 0.4849(4)	0.07546(17), 0.48450(13)
O(2) x,y,z	−0.2890(3),0.2879(3),0.0403(2)	−0.28747(9),0.28798(10),0.03960(7)
wRp, Rp, $\chi^{2}$	4.72% 4.03%, 1.37	2.4% 3.6%, 3.04
Cr, $\mu_{x}$ ($\mu_{B}$)	2.339(16)	0
Cr–O(1) (Å)	1.9721(6)	1.9727(9)
Cr–O(2) (Å)	1.9751(16), 1.9799(16)	1.972(3), 1.982(2)
Pr–O(1) (Å)	2.369(5), 2.298(3)	2.389(7), 2.507(4)
	[3.059(3), 3.104(5)]	[3.046(4), 3.088(7)]
Pr–O(2) (Å)	2.382(3), 2.610(3), 2.712(3)	2.389(7), 2.603(4), 2.729(4)
	[3.286(3)]	[3.281(4)]

There is some ambiguity in the literature over the coordination value of the *A*-site cation in these materials. In ${\text{PrCrO}}_{3}$, the ${\text{PrO}}_{n}$ polyhedra are distorted with Pr–O distances ranging from 2.389 to 2.729 Å if n=8 is assumed, with a maximum Pr–O distance of 3.218(4) Å if n=12. The structural differences in these two different polyhedra are shown in figure 1. For the ${\text{PrO}}_{12}$ unit, the bond lengths are significantly longer than is accepted for a typical Pr–O bond, and the resultant polyhedral shape is highly irregular within 8- and 12-fold coordinated ${\text{PrO}}_{n}$ polyhedra the average values are 2.478 Å and 2.672 Å respectively [20]. It is therefore more physical in this case to assume n=8 coordination. We list some values for both cases, in order to aid comparison with the literature.

${\text{PrCrO}}_{3}$ remains orthorhombic down to the lowest temperature measured, 10 K. The mean volumetric thermal-expansion coefficient was determined to be ${\bar{\alpha }}_{v}=22.4\times 10^{-6}\;K^{-1}$ (calculated over the full temperature range 10–1473 K), comparable to the other orthochromites [21,22]. The system becomes antiferromagnetic below $T_{\text{N}}\approx 240$ K (see electronic supplementary material), with the appearance of a strong magnetic reflection at 4.46 Å. The $Pb^{\prime }n^{\prime }m$ model was fitted against the data, $\Gamma_{3}$ irreducible representation, with a maximum refined moment of $2.34(2)\mu_{b}$ on the ${\mathrm{Cr}}^{3+}$ sites at 10 K along the a-axis. The data were insufficient to fit any moment on ${\mathrm{Pr}}^{3+}$ site, or any canting of the moment on the Cr site, both resulting in divergence of the refinement. Both the ${\text{CrO}}_{6}$ octahedra, and the ${\text{PrO}}_{8}$ polyhedra reduce in volume at a comparable rate, with extremely small changes in the tilt (the M3+ mode is invariant, while the R4+ anti-phase tilt increases slightly to $8.95^{\circ }$) at 10 K. Looking more closely at the octahedral distortion on heating, the R5+ (Cr) mode increases in magnitude by ∼25%, while the X5+ (Cr) mode decreases by only approximately 1%, the X5+ (Pr) mode reduces by approximately 7%. This leads to an overall reduction in the distortion of the Pr-polyhedra, at the expense of some distortion of the Cr-octahedra.

**Figure 2.**
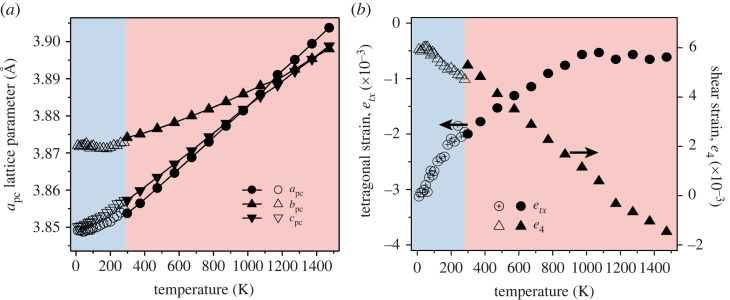
(*a*) Temperature dependence of the lattice parameters of ${\mathrm{PrCrO}}_{3}$ reduced to their pseudo-cubic form (see main text). (*b*) Calculated tetragonal ($e_{tx}$) and shear ($e_{4}$) strain of the system as a function of temperature (the arrows indicate which dataset is plotted on each axis). In both figures, the data below 280 K (blue region) were collected from neutron powder diffraction (open symbols), and those above (red region) were collected using X-ray diffraction (solid symbols). (Online version in colour.)

The symmetry remains orthorhombic up to the maximum temperature considered, 1500 K. Cooperative changes within the material at the atomic scale, results in lattice strains (e) on a macroscopic scale, which are the driving forces for a transition [23]. The spontaneous strains are calculated as $(x_{\mathrm{pc}}-a_{0})/a_{0}$, where $x_{\mathrm{pc}}$ are the pseudo-cubic lattice parameters calculated in the orthorhombic phase ($a_{\mathrm{pc}}$, $b_{\mathrm{pc}}$, $c_{\mathrm{pc}}$), and $a_{0}$ is in this case an isotropically strained lattice parameter that would occur at the same pressure in the high symmetry, cubic phase. Since there is no experimentally observed cubic phase of ${\text{PrCrO}}_{3}$ from which $a_{0}$ can be extrapolated, the expected cubic behaviour is instead estimated from the pseudo-cubic unit-cell volume ($V_{\mathrm{pc}}$) using $a_{0}\approx \sqrt[{3}]{V_{\mathrm{pc}}}$. Figure 2 shows the variation in spontaneous strain $e_{tx}$, and shear strain $e_{4}$, with temperature. There is a crossover in the lattice parameters from (b>a)→(a>b) at 1135 K, which corresponds to a point where the tetragonal strain plateaus, and the shear strain crosses zero. On continued heating, the tetragonal strain remains unchanged, while the modulus of the shear strain begins to increase again. This phenomenon is not unusual in orthorhombic perovskites; while the crossover is usually induced through changes in chemical composition, it is well documented as a temperature effect in ${\text{LaFeO}}_{3}$ [24,25]. The origins of this crossover are the competition between the octahedral tilting, and octahedral distortion. The octahedral rotation may encourage the lengthening of one axis relative to the other, which is then either accommodated or resisted by the in-plane distortion of the octahedra. Since the PXRD data are dominated by scattering from the Pr and Cr, it was not possible to accurately refine the O-positions to investigate the balance between these two effects in ${\text{PrCrO}}_{3}$.

**Figure 3.**
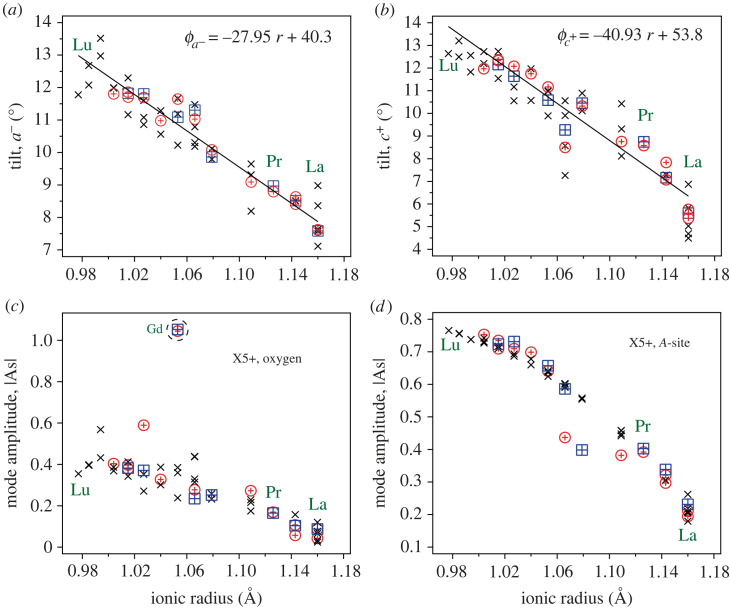
Calculated anti-phase, $a^{-}$ (*a*), and in-phase, $c^{+}$ (*b*), tilt angles plotted as a function of A-site ionic radius for a number of lanthanide orthochromites found in the literature [22,27–50], and from the present study. The ionic radii are compared assuming 3+ oxidation, and eightfold coordination [52]. The lines in both (*a*) and (*b*) are linear best fits to the neutron data at ambient temperature. Also shown are the calculated distortion mode amplitudes for X5+, oxygen octahedra (*c*) A-site cation (*d*). The outlier in (*c*) is for the Gd compound, so it is assumed that absorption corrections have led to an inaccurate structure determination from the neutron data. In all figures, the crosses are calculated from X-ray diffraction data (room temperature), while the squares (low temperature 2–20 K) and circles (room temperature) are calculated from neutron diffraction data. (Online version in colour.)

To compare the high-temperature behaviour with other ${\text{ACrO}}_{3}$ (A=lanthanide) compounds, a similar degree of thermal expansion is observed for A=Nd, with no change in symmetry, and no crossover in the *a*- and *b*-lattice parameters [22]. For A=La, the compound undergoes a phase transition from orthorhombic to rhombohedral symmetry at approximately 550 K, resulting in a discrete drop in the ${\text{CrO}}_{6}$ polyhedral volume (approx. 0.7%) [26]. On continued heating, the volumetric thermal expansion of ${\text{LaCrO}}_{3}$ is comparable with ${\text{PrCrO}}_{3}$. The very small change in tilt angle with changing temperature is typical of the lanthanide orthochromites. To demonstrate this, a large number of reported orthochromite structures from the literature have been compared as a function of *A*-site ionic radius and temperature [22,27–50]. The tilts were calculated consistently through symmetry-adapted basis vector decomposition [11,19,51] allowing for a proper distinction between octahedral tilt, and distortion. This information is summarized in figure 3. While neutron data are more sensitive to oxygen information, a number of structures reported from X-ray measurements are also included in the comparison. It is clear from comparing the high- and low-temperature values determined from neutron diffraction that the tilt angles and polyhedral distortions are relatively insensitive to temperature changes (2–300 K) in all lanthanide orthochromites. Instead, the size of the *A*-site cation dominates the tilt of the oxygen octahedra, varying by $5\text{−}6^{\circ }$ over the series. In all cases, the reduction in *A*-site cation size results in increased levels of tilt, and distortion of both the oxygen octahedra, and the *A*-site polyhedral units, as evidenced by the increase in the X5+ distortion modes. The R5+ (O) and M2+ (O) modes, not shown in the figure, are approximately invariant and close to zero in amplitude. The R5+ (A-site) mode shows a similar increase in amplitude with reduction in cation radius. By contrast, B-site cation size has the opposite influence on the tilts in the series ${\text{LaBO}}_{3}$ (B=Sc, Ti, V, Cr, Mn, Fe, Co, Ni, Cu). There is an approximately linear decrease in the tilts with reduction in B-site cation radius from Sc to Fe [25,53–57], and an eventual raising of symmetry to rhombohedral for the Co-Cu compounds.

### (b) High-pressure structural behaviour

**Figure 4.**
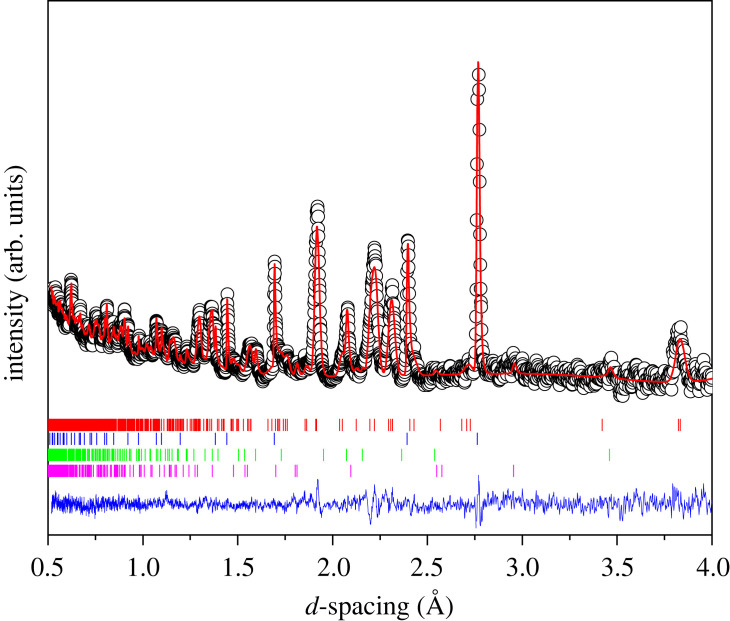
Neutron diffraction pattern and Rietveld refined fit of ${\mathrm{PrCrO}}_{3}$ at 5.6 GPa. The experimental data are shown as open circles, the Rietveld fit as the red line and the blue line is the residual of the fit to the data. The tick marks show the expected positions of reflections from orthorhombic ${\mathrm{PrCrO}}_{3}$ (red), Pb pressure marker (blue), ${\mathrm{Al}}_{2}O_{3}$ (green) and ${\mathrm{ZrO}}_{2}$ (purple), from top to bottom respectively. The ${\mathrm{Al}}_{2}O_{3}$ and ${\mathrm{ZrO}}_{2}$ contribution are from the anvil material used in the high-pressure assembly. (Online version in colour.)

**Figure 5.**
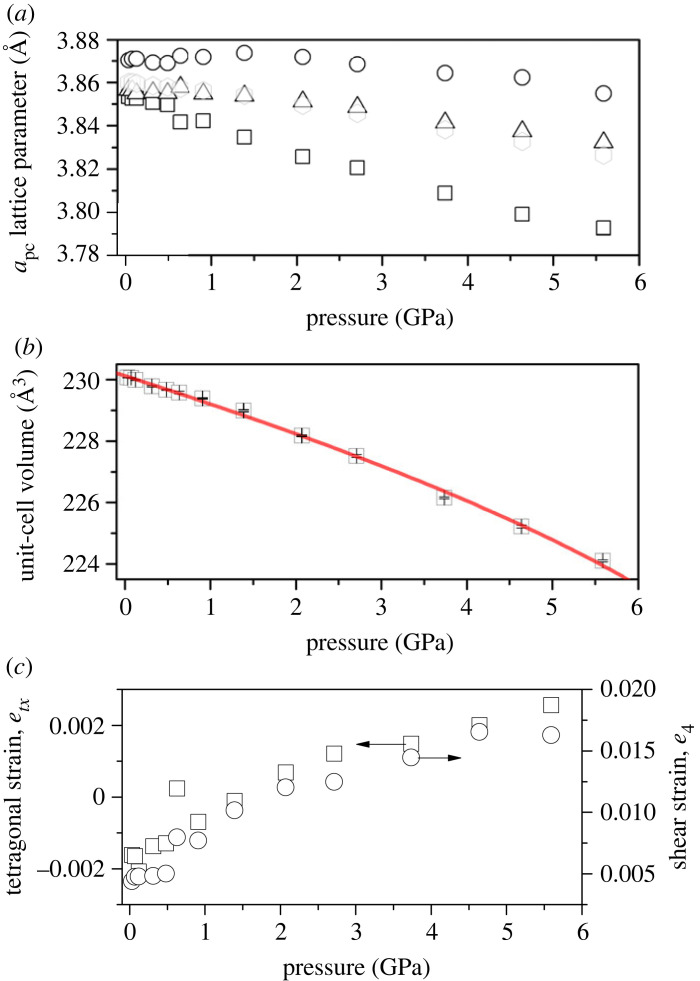
Behaviour of unit-cell parameters of ${\mathrm{PrCrO}}_{3}$ with pressure at 290 K. (*a*) Variation in pseudo-cubic lattice parameter where $a_{\text{pc}}=a_{o}/\sqrt{2}$ (open squares), $b_{\text{pc}}=b_{o}/\sqrt{2}$ (open circles) and $c_{\text{pc}}=c_{o}/2$ (open triangles). The transparent hexagon symbols are the pseudo-cubic lattice parameter ($a_{\text{pc}}$) determined from the orthorhombic unit cell volume ($V_{o}$) as $a_{\text{pc}}={\left(V_{o}/4\right)}^{1/3}$. (*b*) Change in unit-cell volume (squares) with pressure. The third-order Birch–Murnaghan EoS fit to the data is shown as solid red line and determined values given in main text. Error bars are shown but smaller than symbols. (*c*) Determined tetragonal strain ($e_{\text{tz}}$) and shear strain ($e_{4}$) with pressure (see text for details). (Online version in colour.)

Figure 4 shows the neutron powder diffraction pattern and associated Rietveld refinement fit to ${\text{PrCrO}}_{3}$ at 5.6 GPa. The derived structural parameters are in close agreement with those determined at ambient pressure. Upon compression to 5.6 GPa no change in crystal symmetry is observed, only a decrease in all unit-cell parameters, see figure 5. The pseudo-cubic lattice parameter $a_{\mathrm{pc}}$ (parallel to the long Pr–O bonds in the ${\text{AO}}_{6}$ polyhedral unit) is more compressible ($2.72\;{\mathrm{TPa}}^{-1}$) than both $b_{\mathrm{pc}}$ ($0.18\;{\mathrm{TPa}}^{-1}$) and $c_{\mathrm{pc}}$ ($0.92\;{\mathrm{TPa}}^{-1}$). These values are similar to the anisotropic compressibility reported for ${\text{HoCrO}}_{3}$ [58], and similarly for ${\text{GdCrO}}_{3}$, ${\text{EuCrO}}_{3}$ and ${\text{SmCrO}}_{3}$ [59], and ${\text{YCrO}}_{3}$ [60]. However, while the *a*-axis (in the Pbnm setting) for ${\text{NdCrO}}_{3}$ was also found to be significantly more compressible than the other axes, the *b*-axis expanded slightly under pressure [22]. This discrepancy observed in ${\text{NdCrO}}_{3}$ may be linked to some non-hydrostatic components in the applied pressure, evidenced by the presence of peak broadening in the diffraction data [22].

The unit-cell volume variation with pressure is shown in figure 5, and demonstrates a clear negative curvature, which could not be accounted for using a second-order Birch–Murnaghan equation-of-state. Fitting instead to the third-order, the derived values for the bulk modulus ($B_{0}$) and first pressure-derivative ($B^{\prime }$) are 258(3) GPa and −13.89(7), respectively. The value of $V_{0}$ of $230.12(3)\;{\text{Å}}^{3}$ is very close to that extracted from refinement of ambient pressure diffraction data (table 1). The value of $B_{0}$ is significantly different to that calculated from density functional theory [61], though in good agreement with the experimental trend of values from other rare-earth orthochromites [22,58–60,62]. The negative curvature ($B^{\prime }$) of the volume–pressure curve is not seen in the other rare-earth orthochromites, suggesting that the material abnormally softens with pressure. This has, however, been observed in other oxides and perovskites and may be associated with the material exhibiting the onset of ferroelastic properties [63]; similar behaviour has been seen in ${\text{ZrO}}_{2}$ and ${\text{BaTiO}}_{3}$ [64–66]. One explanation is that softening of the material can be a prelude to a phase transition, however, for ${\text{PrCrO}}_{3}$ the softening is observed throughout the pressure range studied. A second explanation may be that there are anomalies in the higher-order elastic constants (presenting as large pressure gradients in shear wave velocities with negative curvature). Such anomalous behaviour may well be a result of ‘soft-mode’ behaviour, often observed in high-symmetry phases near the critical pressure of a second-order phase transition [67].

Figure 5 shows the variation in spontaneous strain $e_{tx}$, and shear strain $e_{4}$, with increasing pressure. This shows that the tetragonal-strain reduces, towards the cubic form (which necessitates, $e_{tx}=0$) albeit with some nonlinear behaviour. At approximately 1.2 GPa, the strain crosses zero, and continues to increase linearly. The *ab*-shear strain, *e*_4_, increases continuously with applied pressure. A simple interpretation of this is that the pseudo-cubic cell changes from being tetragonally compressed to elongated along the orthorhombic *c*-axis, while the increased level of *ab*-shear retains the orthorhombic symmetry through the crossover point.

**Figure 6.**
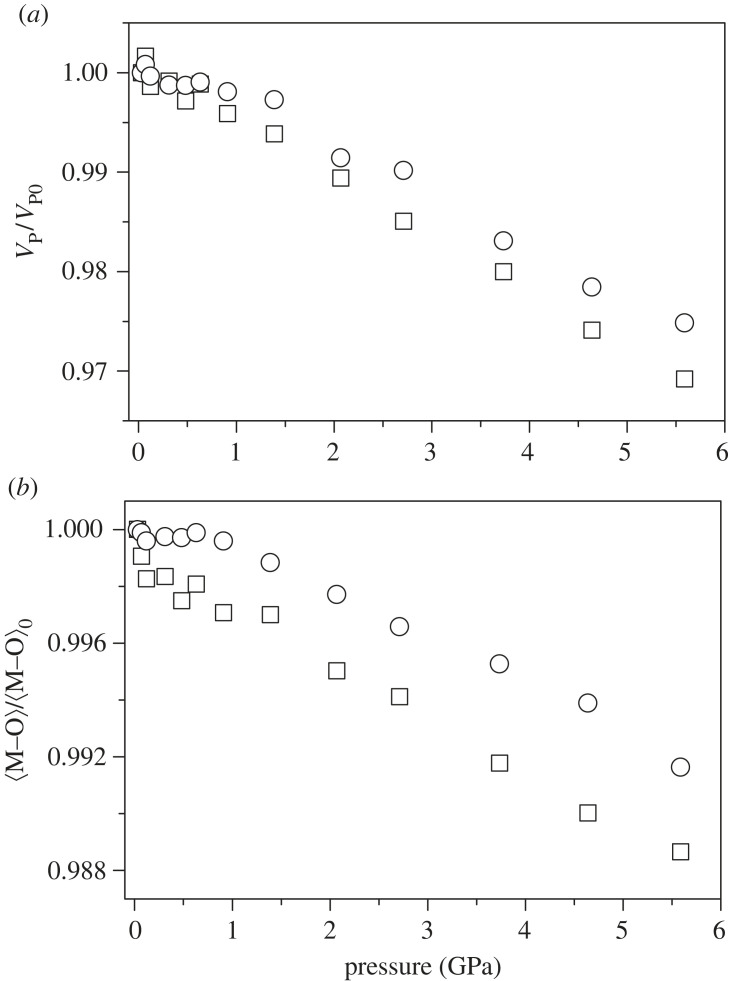
(*a*) Variation in the ${\mathrm{CrO}}_{6}$ (rectangle) and ${\mathrm{PrO}}_{8}$ (circle) polyhedral volumes with pressure in ${\mathrm{PrCrO}}_{3}$. (*b*) Variation in the average M–O bond length (⟨M−O⟩) within the ${\mathrm{CrO}}_{6}$ (rectangle) and ${\mathrm{PrO}}_{8}$ (circle) polyhedra. The values in both (*a*,*b*) are normalized against ambient pressure values.

To better understand how the orthorhombic symmetry is maintained, changes in the tilt and distortion of the polyhedral units should also be considered. The three distinct Cr–O bond lengths within the ${\text{CrO}}_{6}$ octahedra tend to decrease with pressure, with the average of the three (⟨Cr−O⟩) reflecting this behaviour with an approximately linear decrease, see figure 6. The average Pr–O bond in the ${\text{PrO}}_{8}$ polyhedra (⟨Pr−O⟩) is less compressible than ⟨Cr−O⟩, which is also apparent in the polyhedral volume changes also shown in figure 6. The Baur geometric distortion indices of both polyhedra are approximately invariant with increasing pressure [68]. The relative compressibility of the two polyhedra is consistent with the predictions based on the bond valence sum (BVS) ratio, as detailed in the literature [4,69,70]. Based on the ambient pressure structure, the ratio of BVS for the ${\text{PrO}}_{8}$ and ${\text{CrO}}_{6}$ polyhedra is 1.31, which implies that the ${\text{CrO}}_{6}$ octahedra should be more compressible than the ${\text{PrO}}_{8}$ polyhedra, and that the overall structure would be expected to become more symmetric with increasing pressure, consistent with other formally $3^{+}/3^{+}$ (A-site/B-site) charged perovskite systems. The octahedral units show a reduction in tilt angle, with $a^{-}$ reducing from approximately $8.85^{\circ }\to 8.5^{\circ }$, and $c^{+}$ from $8.6^{\circ }\to 8.3^{\circ }$, though still considerably tilted away from the undistorted cubic sub-cell. Additionally, there is a linear increase in the X5+ (Pr) mode by approximately 20% at 5.5 GPa, suggesting that the application of pressure has a similar effect on the distortion of the Pr-O polyhedra as reducing the temperature. The R5+ (O) mode also increases approximately linearly with pressure, related to the *ac*-plane distortion of the ${\text{CrO}}_{6}$ octahedra.

There are very few high-pressure structural studies on the lanthanide orthochromites in the literature, with the majority of them being X-ray diffraction studies, only reporting the changes in lattice parameters [22,58–60]. A neutron study of ${\text{LaCrO}}_{3}$ showed that it was found to change from orthorhombic to rhombohedral symmetry at approximately 5 GPa [62,71,72]. Analysis of the symmetry-adapted basis vectors from the work of Zhou *et al.* [62] shows that the changes in tilt are similarly small to those in ${\text{PrCrO}}_{3}$ over a similar pressure range. However, unlike the changes observed in ${\text{PrCrO}}_{3}$, the $a^{-}$ tilt decreases from $7.76^{\circ }\to 7.59^{\circ }$, while the $c^{+}$ tilt increases from $4.82^{\circ }\to 5.27^{\circ }$. The X5+ (La) and R5+ (La) modes both decrease in amplitude, showing a reduction in La–O polyhedral distortion, while M2+ (O) increases linearly with amplitude. This mode is related to the *ab*-plane distortion of the ${\text{CrO}}_{6}$ octahedra, and isn’t seen to increase with increasing pressure in ${\text{PrCrO}}_{3}$, while the R5+ (O) mode is unchanged with pressure in ${\text{LaCrO}}_{3}$. The reason for these differences is linked to the larger *A*-site ionic radius for La, which results in a less tilted, but more distorted polyhedral system at ambient pressure (figure 3).

The previous study of ${\text{NdCrO}}_{3}$ with pressure only reports changes in lattice parameters with pressure, though the structure reportedly remains orthorhombic up to the maximum 6.4 GPa considered, and the volumetric compression shows no clear softening [22]. ${\text{LuCrO}}_{3}$, ${\text{TbCrO}}_{3}$, ${\text{GdCrO}}_{3}$, ${\text{EuCrO}}_{3}$ and ${\text{SmCrO}}_{3}$ were investigated up to approximately 20 GPa with X-ray diffraction [59]. The authors concluded that larger A-site cation radii lead to the stabilization of a less distorted high-pressure structure (as with ${\text{LaCrO}}_{3}$), whereas smaller *A*-site cations instead accommodate more highly distorted A-site polyhedra, retaining orthorhombic symmetry [59]. This finding seems consistent with what is observed with ${\text{YCrO}}_{3}$ up to 60 GPa [60]. The bulk modulus determined in the present study, is consistent with those determined for the other orthochromites, which shows an approximate trend of a reduction in $B_{0}$ with increases A-site cation radius.

### (c) High-pressure Raman spectroscopy

**Figure 7.**
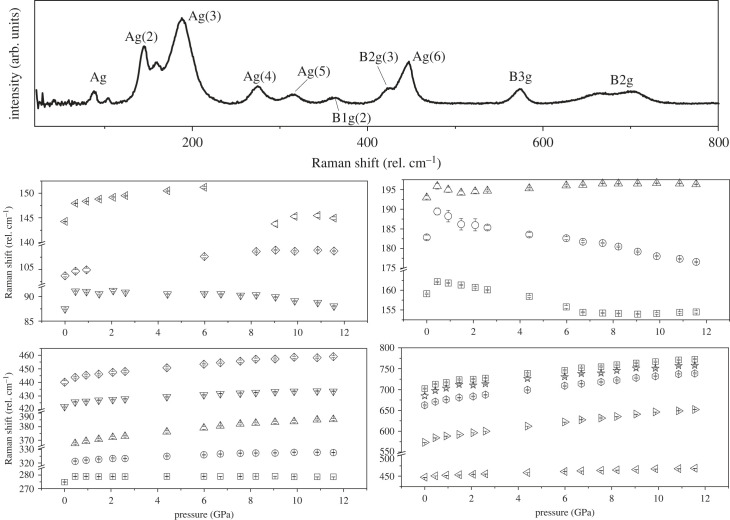
Raman spectra of ${\mathrm{PrCrO}}_{3}$ at ambient conditions, with mode assignments (top). Behaviour of Raman modes of ${\mathrm{PrCrO}}_{3}$ with increasing pressure (bottom). Note that there are breaks in the y-axes of all of the plots.

Figure 7 shows the ambient pressure Raman spectra of ${\text{PrCrO}}_{3}$. Symmetry allows 24 Raman active modes ($7A_{g}+5B_{1g}+7B_{2g}+5B_{3g}$) [73]. There are 16 discernible Raman modes in the spectral region measured, the positions of which are in close agreement with those assigned in previous studies (the assignments are labelled in figure 7). Interestingly, we also observe a mode at ca $160\;{\mathrm{cm}}^{-1}$ which in previous studies could not be assigned, suggested to be a result of an impurity however, no such impurity is observed in the neutron diffraction data [73]. The modes above $600\;{\mathrm{cm}}^{-1}$ do not correspond to normal Raman modes expected of the Pbnm orthorhombic structure. However, these may be ascribed to some form of local lattice imperfection and could be formed by distorted ${\mathrm{Cr}}^{4+}{\text{O}}_{6}$ and/or ${\mathrm{Cr}}^{4+}{\text{O}}_{n}$ (n≠6) [74]. Similarly, this is suggested to occur in ${\text{LaCrO}}_{3}$ which is thought to be cation-deficient ${\text{La}}_{1-y}{\text{CrO}}_{3}$ and ${\text{LaCr}}_{1-x}A_{x}{\text{O}}_{3}$ (A=Co,Ni) [74]. In the octahedral structure, ${\mathrm{Cr}}^{4+}$ has partially filled $t_{2g}$ orbitals in which a Jahn-Teller distortion affecting the local environment of the ${\mathrm{Cr}}^{4+}$ may be present [74]. Given that the unassigned mode at ca $160\;{\mathrm{cm}}^{-1}$ is observed to a similar extent in the previously reported studies, it may be a result of local symmetry breaking, rather than chemical impurity [73].

The majority of the observed modes shift to higher energy with increasing pressure (figure 7). However, in particular, the $B_{2g}$ and $A_{g}$ modes are found to soften and are related to rotational modes and some small discontinuous behaviour is observed in these and other modes around 7 GPa. Such behaviour may be indicative of a phase transition, at pressures beyond that considered in the diffraction study.

## 4. Conclusion

Neutron and X-ray diffraction has been used to fully characterize the structure of ${\text{PrCrO}}_{3}$ as a function of pressure and temperature. The variable temperature data show that the sample remains orthorhombic over the full temperature range considered (10–1500 K), with a crossover in the magnitude of the *a*- and *b*-lattice parameters at approximately 1135 K, driven by competition between the tilt and distortion of the oxygen octahedra. In both the temperature and pressure studies, the changes in the octahedral tilts were found to be small, not changing by more than approximately $0.1^{\circ }$ and $0.3^{\circ }$, respectively, while the relative unit-cell strains changed more substantially. In comparison with other lanthanide orthochromites, the induced structural changes are relatively small, suggesting that A- or B-site doping can induce significantly larger effects than the temperatures or pressures considered in this study.

The high-pressure data collected at ambient temperature show an unusual softening in the unit-cell volume, which we suggest may be a prelude to a second-order phase transition at higher-pressures still, which may also be apparent in the high-pressure Raman spectra at ca 7 GPa. As both La and Pr have relatively large ionic-radii (within the lanthanide series), it is expected that this transition would be similar in nature to that observed in ${\text{LaCrO}}_{3}$ [62]. Whether a similar trend exists across the series remains unknown, though a future study with a more highly distorted ambient structure would be interesting for comparison. With this in mind, it seems likely that the Néel temperature of ${\text{PrCrO}}_{3}$ will shift to higher temperatures with applied pressure, though further experimental data are required to verify this [75].

## Data Availability

Data supporting this study are openly available from the STFC. DataGateway at the DOIs referenced here [76–78]. The data are also provided in electronic supplementary material [79].
